# Reliability between measurements obtained by caliper and digital photography in facial anthropometry

**DOI:** 10.1590/2317-1782/e20250193en

**Published:** 2026-03-31

**Authors:** Rayssa Silva Santos Andrade, Paulo Vitor de Oliveira Santos Matos, Maria Inês Beltrati Cornacchioni Rehder, Raphaela Barroso Guedes-Granzotti, Kelly da Silva, Carla Patrícia Hernandez Alves Ribeiro César

**Affiliations:** 1 Departamento de Fonoaudiologia, Universidade Federal de Sergipe – UFS - São Cristóvão (SE), Brasil.; 2 Pós-graduação em Perícia Fonoaudiológica, CEFAC – Saúde Educação - Rio Claro (SP), Brasil.; 3 Parole Fonoaudiologia Clínico Científica - Rio Claro (SP), Brasil.; 4 Departamento de Fonoaudiologia, Universidade Federal de Sergipe – UFS - Lagarto (SE), Brasil.

**Keywords:** Anthropometry, Face, Speech Therapy, Forensic Sciences, Methodology

## Abstract

**Purpose:**

To investigate the reliability between direct and indirect measurements in facial anthropometry.

**Methods:**

The sample consisted of 60 subjects aged between 16 and 48 years. After applying the eligibility criteria, indirect objective anthropometric facial examinations (SAPO® and IMAGEJ® software) and direct frontal anthropometric examinations were performed using a digital caliper at the anthropometric points: glabella, sub nasale, gnathion, alar, exocanthus, and chilling, with the patients seated in a comfortable position and head in a natural position. Two trained and calibrated examiners performed the measurements three times to obtain the arithmetic mean. The participants were photographed to perform the indirect measurements. The results were analyzed using descriptive statistics (measures of absolute and relative frequency and summary measures of central tendency and dispersion) and inferential statistics (Chi-square test, Bland-Altman, paired t-test, and Intraclass Correlation Coefficient), considering a significance of 5%.

**Results:**

There was intra- and inter-observer agreement, sexual dimorphism (female measurements smaller), higher values of direct anthropometry when compared with photoanthropometry, and differences between the software used and the caliper with ImageJ®.

**Conclusion:**

There was a divergence between direct and indirect anthropometries, which do not represent the same values and proportions, depending on the anthropometric point used, and the professional should consider this aspect. Facial anthropometric comparisons should be carried out using the same software in order to avoid divergences in analyses.

## INTRODUCTION

In Orofacial Myofunctional Therapy, measuring orofacial structures makes it possible to understand the contribution of form and its relationship to function, and as well in the reverse^(1)^. For forensic purposes, photographic documentation aids in human identification and forensic reconstruction. It is considered the gold standard because it allows for the collection of additional information whenever necessary, particularly during the review of criminal cases, and is considered a simple, inexpensive, and accurate method^(2)^. Thus, it can be performed with a single camera, producing two-dimensional (2D) images, or with multiple cameras or by facial scanning, enabling the generation of three-dimensional (3D) images^(3)^.

In this way, facial anthropometry objectively measures and compares dimensions between individuals, using anatomical reference points^(4)^. For direct assessment, calipers may be used, whereas for indirect assessment, photographs can be taken and measurements obtained through software. The freely available postural assessment software (SAPO®) can be used for the indirect measurement of facial features in speech-language forensic evaluations^(5)^. However, another free program that can be used in forensic investigations, IMAGEJ®, is a digital tool that offers scalability, as plug-ins make it possible to add algorithms for image analysis^(6)^. However, training is urgently required since the results of the measurements obtained depend on the expertise of the user or clinician^(7)^.

It should be noted, however, that in relation to software use, the literature indicates that, although computerized facial analysis provides greater analytical capabilities and precision, errors such as false positives or false negatives may still occur^(8)^. It is therefore evident that, due to the limited number of studies addressing the reliability of direct measurements (using calipers on the patient's facial skin tissue) and indirect measurements, with the various software programs employed in facial anthropometry within Speech-Language Pathology, it is necessary to verify the feasibility of their standardized use, supported by scientific evidence for professional application.

Therefore, this study aims to investigate the reliability of direct and indirect measurements in facial anthropometry.

## METHOD

This cross-sectional, descriptive, and observational study was approved by the Research Ethics Committee of the Federal University of Sergipe (CAAE No. 78821524.9.0000.5546 and Process No. 6.823.063). All participants provided a written Informed Consent Form.

The sample was obtained by convenience and comprised of 60 participants, aged between 16 and 48 years (mean: 23.98, SD: 7.47), with 30 (50%) being male and 30 (50%) female, all considered healthy. It should be noted that a sixteen-year-old adolescent was included to complete the sample, as the number of participants was determined by calculating the sample size using the Intraclass Correlation Coefficient (ICC), with a minimum acceptable value of 0.75 and an expected ICC of 0.90, a significance level of 5% (α = 0.05), and a statistical power of 80% (1–β = 0.80), resulting in the aforementioned value. To avoid confounding bias regarding this participant's involvement in the study, the ICC was recalculated after excluding her for the sample, and the result remained unchanged, thereby allowing her to be retained in the study.

The inclusion criteria were as follows: age, harmonious dental occlusion (first molar relationship in mesial step and balanced maxillomandibular relationship), nasal breathing mode (by the possibility of exclusive nasal use for more than two minutes, measured with a stopwatch); and orofacial motor skills screening score (OMS) of up to one point^(9)^. The exclusion criteria were: occlusal alterations (Angle Classes II and III, malocclusions, open bite, crossbite and overbite); oral breathing pattern (due to complaint or previous treatments); positive history for neurological disorders affecting mobility and/or muscle tone; motor alterations that could interfere with body stability and, consequently, with stability during procedures; ongoing treatment (orthodontic and/or speech therapy); plastic surgeries and other structural alterations in the cranio-oro-cervical complex of different origins (hereditary, genetic or environmental, such as facial trauma and burns).

Images that did not meet the criteria for good or excellent quality, according to the adopted classification^(10)^, were reshot or deleted when retaking was not possible.

For the application of eligibility criteria, participants were screened using the OMS^(9)^. Those with a score equal to or greater than two points were referred for a complete evaluation in the respective area and excluded from the study. Subsequently, objective anthropometric facial examinations were performed using a Digital Caliper and two software programs: SAPO^®^ and IMAGEJ^®^.

The procedures were conducted in a quiet and private environment, at a predetermined time, and by mutual agreement with the participant. At that time, information regarding identification and socioeconomic data was collected, and the research procedures described below were applied.

**Figure 1:**
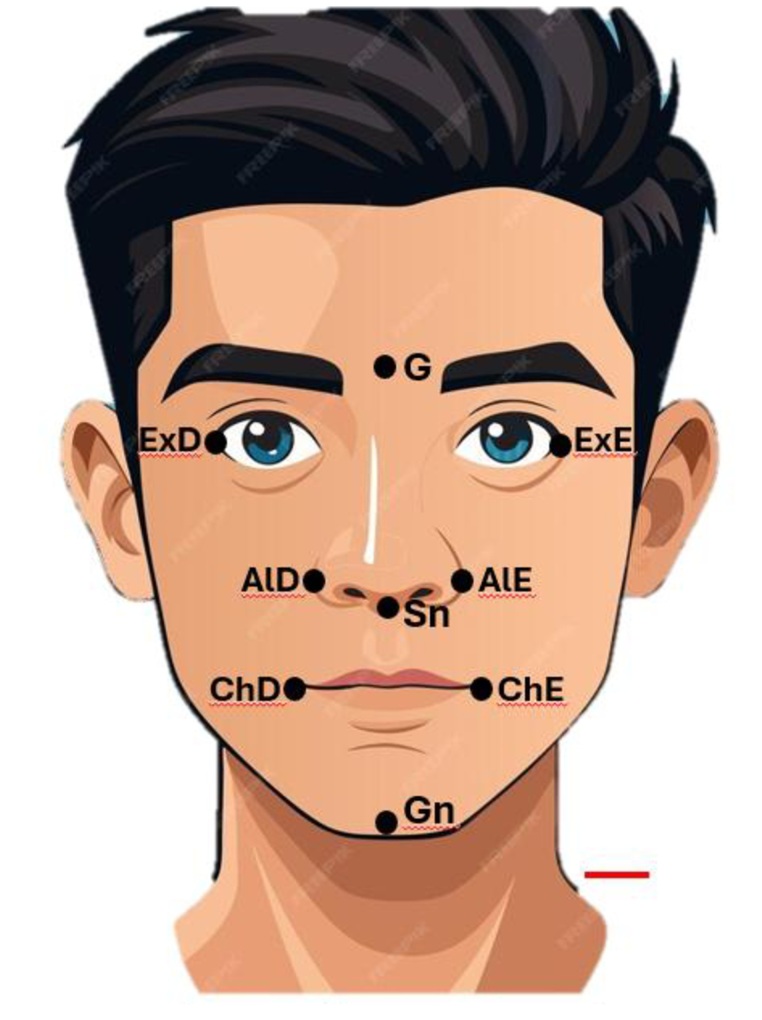
Anthropometric landmarks applied in this study, both in direct and indirect anthropometry

For direct objective assessment, a digital caliper was used, and reference lines were drawn on the face (previously sanitized with alcohol) with the aid of a dermatographic pencil. Measurements were obtained with a precision of 0.01 mm, applying the least possible pressure to the participants’ skin surface. Nine anthropometric points were marked: Glabella (G), Subnasale (Sn), Gnathion (Gn), Outer Canthus of the Eye (Ex), and Alare (Ald-Ale), in order to determine the middle (G-Sn) and lower (Sn-Gn) facial thirds, as well as the distance from the outer canthus of the right eye to the right commissure of the lip (ExD-ChD) and from the outer canthus of the left eye to the left commissure of the lip (ExE-ChE), as shown in Figure 1.

Caption: Glabella (G), Subnasale (Sn), Gnathion (Gn), Outer Canthus of the Eye (Ex), Alae of the Nose (Ald-Ale)

Note: the horizontal red line is 10 mm in length and was introduced to parameterize measurements in the photoanthropometric tests performed

The measurements were taken three times by two independent evaluators in order to obtain arithmetic means. It is important to note that the evaluators had been previously trained for this purpose, through a 14-hour theoretical-practical course, that culminated in an assessment involving facial measurements using both direct and indirect anthropometry. The results obtained were compared with those of one of the authors, who was considered the gold standard in the field due to her professional expertise in orofacial motor skills assessment, her teaching experience in the area, and her doctoral degree. Only after this stage, referred to as evaluator calibration, was the anthropometric analysis conducted.

For the photographs and direct anthropometry, all participants remained seated in a chair without head support, with their feet on the floor, without glasses or makeup, hair tied back, head positioned in the Frankfurt horizontal plane, dental occlusion respecting the free functional space (without contact between the occlusal cusps), and against a white background. Participants were instructed to maintain a neutral facial expression (without contractions). The ambient lighting was considered satisfactory for the photographs. The results were recorded on a sheet specifically for this purpose. It should be noted that asymmetry between the hemifaces was defined as values equal to or greater than 2 mm^(11,12)^, across all measurement methods.

Next, photographic records (2D photographs) of the participant were obtained with the anthropometric points marked on the skin. The camera function of a Samsung Galaxy S23® Android smartphone was used in "face" mode (in this mode, the subject is framed within a white square to improve the standardization of facial positioning). A previous study^(13)^ has shown that digital tools embedded in smartphones allow for high-quality image capture for facial measurement and, therefore, for clinical use, and that non-professional equipment can be employed in photoanthropometry^(14)^. The device was fixed to a tripod, positioned 1.5 m away from the participant, and frontal photographs were taken. The images were then saved to a computer and opened in two software programs for analysis: SAPO® and IMAGEJ®, in order to measure the same facial points and verify whether the proportions of the measurements obtained using the caliper were maintained using the selected software.

To this end, the images were inserted into Microsoft® PowerPoint and adjusted in size and rotation. Then, 10mm horizontal and vertical lines were introduced (using the software's drawing tool and verifying the dimensions in the measurement window that appears while drawing the line). These lines were then added to the images to standardize the measurements in both software programs, horizontally and vertically. The same measurements were taken three times, and the arithmetic mean was calculated. The results were then transferred to a record form. To verify intra-observer agreement, two weeks after obtaining the initial photoanthropometric measurements (to avoid recall bias and reduce fatigue from previously performed measurements, thereby ensuring intra-examiner reliability), twelve photographs were remeasured again in a randomized manner in each software tested. If intra- and inter-observer agreement was confirmed, data collection proceeded.

Using the values obtained from the anthropometric points, a numerical facial analysis of the facial thirds and hemifaces was performed. This analysis indicated whether they were larger, smaller, or equal, recording the larger side (in the case of hemifaces) and the larger facial third, according to the MBGR^(15)^ protocol.

The same measurements were taken three times, and the arithmetic mean was calculated. The results were transferred to a data collection form. At the end of the data collection, the data were organized into spreadsheets in Microsoft Office Excel 2013 (Microsoft® Corp., Redmond, WA, USA). The normality of the data was verified with the Shapiro-Wilk test. The analysis of the results was performed using descriptive statistics (frequencies, means, and standard deviations) and the Bland-Altman concordance technique^(16)^.

Intra-observer agreement was assessed using the paired t-test, and inter-examiner agreement was assessed using the Intraclass Correlation Coefficient (ICC). The analysis was performed on twelve randomly selected images (using Microsoft® Excel) 30 days after the initial measurements, corresponding to 20% of the sample (12 new measurements for each evaluator). It is important to note that these analyses were performed only on indirect anthropometric measurements, due to the difficulties of repeating in-person measurements required for direct anthropometry. The numerical facial analysis was subjected to statistical analysis using the Equality of Proportions test. All analyses were conducted with JAMOVI software. A significance level was adopted when the p-value was less than 5%.

## RESULTS

To verify intraobserver agreement (paired t-test and ICC), the measurements taken by evaluator 1 after 30 days in 20% of the sample revealed no statistically significant differences using direct and indirect methods, as can be seen in Table 1.

**Table 1:** Statistical analyses (paired t-tests and ICC) of intra-observer agreement in the two software programs employed

Evaluator	Software	Stat. Test	Middle third	Lower third	AlarR - AlarL	Hemiface R	Hemiface L
**Evaluator 1**	SAPO®	Paired t-test	0.128	0.286	0.554	0.075	0.610
		ICC	0.999	0.997	0.999	0.998	0.999
	IMAGEJ®	Paired t-test	0.924	0.976	0.886	0.820	0.292
		ICC	1.000	0.999	0.999	0.999	1.000
**Evaluator 2**	SAPO®	Paired t-test	0.323	0.970	0.632	0.799	0.548
		ICC	0.993	0.995	0.990	0.996	0.995
	IMAGEJ®	Paired t-test	0.008	0.345	0.838	0.722	0.439
		ICC	0.997	0.999	0.996	0.999	0.999

**Caption:** R = right, L = left, ICC = Intraclass Correlation Coefficient, Stat. Test=Statistical Test. **Source:** authors

The analysis of interobserver agreement using the ICC test, based on the averages obtained from the anthropometric points, revealed that the measurements taken by evaluator 1 and repeated by evaluator 2 were consistent with each other, When using both the SAPO® software and IMAGEJ®, as shown in Table 2. The ICC was greater than 0.9, indicating excellent agreement and consistency in the measurements performed by the evaluators.

**Table 2:** Analysis of inter-observer agreement between Evaluator 1 and Evaluator 2 in the two software programs employed

ICC	Softwares	MT E1	MT E2	LT E1	LT E2	Al-Al E1	Al-Al E2	HFR E1	HFR E2	HFL E1	HFL E2
	**SAPO®**	0.981		0.987		0.98		0.983		0.976	
	**ImageJ®**	0.992		0.999		0.986		0.996		0.993	

**Caption:** E1= Evaluator 1, E2 = Evaluator 2, Al=alare, R = right, L= left, HF=hemiface (measure of exocantum a cheilion), LT= Lower third, MT = Middle third, Statistical test applied: Intraclass Correlation Coefficient (ICC). **Source:** authors

Comparative analysis of the averages obtained by the methods used showed that the direct measurements were significantly different and larger than those obtained by 2D photoanthropometry, with the direct method being considered different from the photoanthropometries according to the statistical test used, the Bland-Altman test. When comparing the photoanthropometric measurements from the software used, the only difference observed in females was in the middle third of the face, whereas in males the differences were found in both the middle and the lower thirds. Thus, the photoanthropometry obtained by SAPO® and IMAGEJ® are partially comparable, depending on the anthropometric point analyzed and the sex of the participant (Table 3).

**Table 3:** Measurements obtained through direct use of a digital caliper (direct evaluation) and through software applications (SAPO® and IMAGEJ®)

Sex/Results		Nasal ala			Hemiface R			Hemiface L			Middle third			Lower third		
		Cal.	SAPO®	IMAGEJ®	Cal.	SAPO®	IMAGEJ®	Cal.	SAPO®	IMAGEJ®	Cal.	SAPO®	IMAGEJ®	Cal.	SAPO®	IMAGEJ®
F.	Mean	33.5	23.3	23.7	69.3	41.0	41.4	68.5	40.9	41.3	56.9	38.5	39.2	59.7	37.4	37.6
	Median	33.5	23.5	23.4	70.2	40.6	41.0	68.3	40.7	41.3	54.3	38.5	38.3	60.5	37.8	37.7
	SD	3.8	2.54	2.8	3.86	4.96	5.21	3.92	4.68	5.11	5.72	6.23	7.09	6.74	4.11	4.18
	Minimum	26.2	18.5	18.0	60.1	31.0	30.4	58.7	30.8	30.1	46.9	27.4	26.8	47.4	28.7	28.9
	Maximum	39.8	29.5	30.5	76.2	49.3	50.8	75.1	49.6	51.7	68.5	52.2	52.8	70.2	44.5	44.3
	Bland-Altman	Cal. and SAPO®: t = -13, df = 29, p-value = 2e-13: Cal. and IMAGEJ®: t = 12, df = 26, p-value = 2e-12: SAPO® and IMAGEJ®: t = -1, df = 26, p-value = 0.3.			Cal. and SAPO®: t = 29, df = 29, p-value = <2e-16; Cal. and IMAGEJ®: t = 29, df = 29, p-value = <2e-16; SAPO® and IMAGEJ®: t = -1.7, df = 29, p-value = 0.1.			Cal. and SAPO®: t = 29, df = 29, p-value = <2e-16; Cal. and IMAGEJ®: t = 28, df = 29, p-value = <2e-16; SAPO® and IMAGEJ®: t = -2, df = 29, p-value = 0.06			Cal. and SAPO®: t = 16, df = 29, p-value = 7e-16; Cal. and IMAGEJ®: t = 14, df = 29, p-value = 1e-14; SAPO® and IMAGEJ®: t = -0.28, df = 29, p-value = 0.01.			Cal. and SAPO®: t = 22, df = 29, p-value = <2e-16; Cal. and IMAGEJ®: t = 24, df = 29, p-value = <2e-16; SAPO® and IMAGEJ®: t = -0.83, df = 29, p-value = 0.4.		
M.	Mean	36.9	24.5	24.6	72.9	41.0	40.8	72.1	40.8	41.0	56.9	36.5	37.1	64.8	41.8	37.1
	Median	37.2	24.9	24.9	73.7	41.8	41.6	72.3	41.6	41.2	57.5	37.0	37.1	66.4	41.2	37.1
	SD	3.21	2.82	3.18	4.61	4.59	5.72	3.95	4.63	4.99	5.28	6.39	7.28	7.3	4.85	7.28
	Minimum	30.7	19.4	19.2	62.4	29.4	24.2	64.9	28.6	27.9	45.6	25.7	25.3	37.0	31.4	25.3
	Maximum	43.0	29.8	31.1	79.6	49.2	49.0	77.8	47.9	50.2	71.5	49.1	49.5	77.0	52.3	49.5
	Bland-Altman	Cal. and SAPO®: t = 15, df = 29, p-value = 3e-15; Cal. and IMAGEJ®: t = 13, df = 29, p-value = 7e-14; SAPO® and IMAGEJ®: t = -0.69, df = 29, p-value = 0.5.			Cal. and SAPO®: t = 35, df = 29, p-value = <2e-16; Cal. and IMAGEJ®: t = 22, df = 29, p-value = <2e-16; SAPO® and IMAGEJ®: t = 0.15, df = 29, p-value 0.9.			Cal. and SAPO®: t = 33, df = 29, p-value = <2e-16; Cal. and IMAGEJ®: t = 30, df = 29, p-value = <2e-16; SAPO® and IMAGEJ®: t = -0.96, df = 29, p-value = 0.3.			Cal. and SAPO®: t = 17, df = 29, p-value = <2e-16; Cal. and IMAGEJ®: t = 14, df = 29, p-value = 8e-15; SAPO® and IMAGEJ®: t = -2,2, df = 29, p-value = 0.03.			Cal. and SAPO®: t = 19, df = 29, p-value = <2e-16; Cal. and IMAGEJ®: t = 18, df = 29, p-value = <2e-14; SAPO® and IMAGEJ®: t = 4.4, df = 29, p-value = 1e-0.4.		

**Caption:** df = degrees of freedom, F = female, M = male, Cal. = caliper, SD = standard deviation, t=trend, Bland-Altman Statistics^(14)^. **Source:** authors

Table 4 presents the convergences of the numerical facial analyses between the thirds of the face in the direct and indirect evaluations, which did not reveal any significant differences according to the statistical test applied. However, when the proportions of the hemifaces were compared, statistically significance differences were observed, indicating divergences across all methods used in this study.

**Table 4:** Comparative numerical facial analysis between digital calipers and photoanthropometric measurements (SAPO® and IMAGEJ®)

Analysis Variable	Method	Classification			Total	P-value
		No. of participants with > LT	No. of participants with < LT	No. of participants with = LT		
**Facial thirds**	IMAGEJ®	30	20	10	60	0.254
	Caliper	42	12	6	60	
	SAPO®	34	16	10	60	
	**Total**	106	48	26	180	
		No. of participants with > HFR	No. of participants with < HFR	No. of participants with = HF	Total	P-value
**Hemiface**	IMAGEJ®	05	02	53	60	<0.001
	Caliper	20	10	30	60	
	SAPO®	05	01	54	60	
	**Total**	30	13	137	180	

**Caption:** R = right; Inf.=inferior; No. = number; HF=hemiface (measure of exocantum a cheilion); LT= Lower third; MT = Middle third; > = greater than, < = less than; = equal. Statistical test applied: Chi-square test. **Source:** authors

In Table 5, based on the measurements taken with the caliper, it was possible to observe sexual dimorphism in the averages obtained from the anthropometric points alD-alE, Sn-Gn, ExD-ChD, and ExE-ChE, with the exception of the middle third of the face. Overall, female facial measurements were smaller than male measurements, according to Student's t-test.

**Table 5:** Comparison of mean measurements obtained with calipers (direct anthropometric assessment) between males and females

Results/Proportions	Alare		Hemiface R		Hemiface L		Middle Third		Lower Third	
	M.	F.	M.	F.	M.	F.	M.	F.	M.	F.
**Mean**	36.9	33.5	72.9	69.3	72.1	68.5	56.9	56.9	64.8	59.7
**Median**	37.2	33.5	73.7	70.2	72.3	68.3	57.5	54.3	66.4	60.5
**SD**	3.21	3.8	4.61	3.86	3.95	3.92	5.28	5.72	7.3	6.74
**p-value**	<0.001		0.002				0.001			

**Caption:** F. = female; L = left; M. = male; R = right; SD = standard deviation. Statistical test applied: Student's t-test. **Source:** authors

## DISCUSSION

The aim of this study was to investigate the reliability between direct and indirect measurements in facial anthropometry in Brazilian adults, in order to compare the methodological criteria applied both in facial assessment and in clinical practice in speech-language pathology. In this way, the study seeks to provide scientific evidence that can support professionals in choosing one method over another. Researchers^(17)^ stated that studies in the health field should establish and document validity, as well as verify the reliability of the measurement instruments applied in professional practice. Confirming the above, researchers^(18)^ emphasized that comparing categorical variables is a frequent requirement in health studies and that such comparisons may lead to different inferential conclusions depending on the analysis method applied, reinforcing that the choice of analytical technique requires solid theoretical grounding and that the parameters of use must be clearly described.

Regarding the topic of this study, facial anthropometry can be performed using different resources, with the one used in photographs (called photoanthropometry) offering some advantages, such as being non-invasive, low cost, fast, reproducible, and allowing images can be archived for future comparisons of results^(19)^. However, the literature also reports certain limitations. The first, according to the literature^(20)^, is that represents a two-dimensional representation of a three-dimensional reality, it may constrain the professional approach. Another is that essential details can be lost during image exportantion^(19)^. There is also the possibility of image artifacts, deformations (whether postural or equipment-related) which may compromise morphological accuracy and thereby reduce the credibility of the analysis^(21)^.

Another aspect to be emphasized is the possibility that the presence of emotional facial expressions may significantly affect the process of facial images comparison. In light of this, one of the measures adopted in this study was to instruct participants to maintain a neutral facial expression, thereby avoiding distortions in both direct and indirect assessments. Furthermore, the reinforcement of previously marked facial landmarks in the photographs sought to ensure consistency in measurement, since even slight positional variations, whether upward or downward, could independently generate discrepancies between the study methods.

A procedure is considered reliable when it consistently yields the same result, within acceptable variations, across repeated measurements of the same variable^(22)^. Accordingly, a study^(23)^ described the postural assessment software (SAPO®) as a reliable tool for postural analysis, since inter-rater agrément was as very good to excellent. Moreover, regarding repeatability factor, the data also indicated good intra-rater reliability.

Nevertheless, the authors considered prior experience with the software to be a determining factor for greater reliability. It should be noted that this software is commonly employed in Physiotherapy, with no published studies in Speech Therapy, thereby justifying the present investigation. Regarding IMAGEJ®, several studies have utilized the software in human facial identification, including research on the use of iris diameter as a reference unit in digital photography^(24)^, comparisons of facial areas before and after rhytidoplasty^(25)^, assessments of facial regions before and after treatment with 0.1% tacrolimus ointment for facial vitiligo^(26)^ and applications in forensic anthropometry for sex estimation, for example^(27)^.

Regardless of the method employed, measurements taken three times at each anthropometric reference point enable for intra-examiner agreement. This implies that, once a method is adopted, comparisons can be performed provided that the same principles and techniques are respected, thereby favoring data replicability. Nevertheless, the values obtained in direct assessment were higher than those obtained through photoanthropometry, using the technique applied, irrespective of sex or the anthropometric point analyzed. This occurred because the caliper allows for direct dimensioning of the reference points and can be considered a standard in studies involving soft tissues. Furthermore, previous studies have demonstrated that the difference between cephalometric points and those measured with the caliper in soft tissue averaged between 1.2 mm and 1.5 mm for the lower and upper thirds, respectively, which justifies repeated measurement to minimize analytical errors^(28)^. Another study^(29)^, which evaluated facial type in children, reported only slight agreement between the cephalometric method and direct caliper measurements, indicating that caliper-based measurement is a viable alternative for clinical practice in orofacial motor skills.

Even with a prior calibration of ten millimeters (one centimeter) to standardize results across software programs, the measurements obtained remained dimensionless, i.e., expressed in pixels. To minimize this bias, when comparing direct and indirect measurements, it is inferred that additional techniques should be applied, such as the use of facial indices (by dividing a smaller facial dimension by a larger one and multiplying the result by 100; for example, en-en/al-al), as suggested by researchers^(14)^. It should be noted that indices were not the focus of this study but are proposed as a recommendation for future investigations.

When comparing facial measurements, both direct and indirect (obtained with SAPO® and IMAGEJ® software), discrepancies were observed across methods and between sexes, with the middle third being the most asymmetrical in both sexes and the lower third only for males. Study^(11)^ demonstrated that anthropometric landmarks located in the lower third of the face exhibit a higher asymmetry index than those in other regions, even in 3D image evaluation, and this aspect should be taken into account. The researchers^(11)^ also emphasized that, even in faces classified as asymmetrical, certain measurements did not reveal differences in the recorded values. Thus, further studies are required to establish reliable reference values for analysis and clinical management.

The researcher added that small differences (between 3-4mm for linear analyses and 3°-4° for angular analyses) are not recognized as asymmetries. They would be considered functional asymmetries. However, in functional analyses of the smile, for example, differences of 3mm are sufficient for the asymmetry to become visible and not perceived as aesthetically pleasing, possibly representing a pathological asymmetry^(30)^. In view of the above, the non-application of the asymmetry index proposed in the literature is regarded as a limitation of the present study^(31)^.

However, it can be inferred that even when statistically significant differences are present, they may not translate into clinical relevance, particularly in cases of discrepancies smaller than 2.0 mm. This is due to variability in intra- and inter-rater measurements, with such differences being considered minimal, as well as the possibility of individual anatomical variations. It should be emphasized that, in professional practice, prior calibration (in this study: 10 mm) is essential for photoanthropometry, and direct anthropometric measurements must be replicated three times to obtain a mean value, thereby ensuring that the measurements are systematic, replicable, and consequently more precise.

Another aspect to be highlighted is that the software programs demonstrated compatibility in the values obtained for females in Al-Al, ExD-ChD, ExE-ChE, and Sn-Gn, and for males for Al-Al, ExD-ChD, and ExE-ChE. These findings should be taken into account when employing software in both clinical and forensic practice. Factors such as skin reflectance may interfere with the accuracy of facial recognition systems, depending on the quality of image acquisition, and this variable should be considered during indirect anthropometric analyses.

Based on the results obtained, it can be stated that, depending on the anthropometric landmarks analyzed, different software programs can be employed in photoanthropometry, and that measurements of the hemifaces and the nasal alae, irrespective of sex, can be considered reliable. The use of SAPO® stands out as software to facial anthropometry by speech-language pathologists, both in clinical practice and in binary facial comparison within speech-language pathology expertise. The same applies to IMAGEJ®, which, according to the literature^(32)^, has been used in research due to its versatility, accessibility, and open source availability, and is also extensively applied in forensic cases.

The Federal Police, for example, employs SAFF-2, a proprietary system (not available as open source), which demonstrates precision in photoanthropometric analyses for most of the measurements used in the research comparing anthropometric landmarks (out of a total of 27 points, 21 were concordant) between the frontal and lateral norms of the participants^(33)^. Currently, stereophotogrammetry has been the preferred choice, as it enables a three-dimensional view of a reformatted 2D image, qualifying it and making it more realistic, according to the literature^(34)^. Nevertheless, despite its advantages, the technology that allows the capture of 3D facial images, using multiple cameras or facial scanners at different angles to provide facial depth, is not yet widely disseminated. In this context, 2D photographs captured with a single camera, as used in photoanthropometry, remain necessary and represent a more accessible resource.

It should also be noted that facial measurements obtained from 2D images may be either underestimated or overestimated to varying degrees, depending on the angle and orientation of each image, when compared with three-dimensional facial measurements. Therefore, for purposes of human identification, it is recommended that measurements derived from two-dimensional images be analyzed individually and interpreted with caution, taking into account the specificities of the angle and orientation of the image used, according to the literature^(35)^, this constitutes a limitation that must be acknowledged.

Numerical facial analysis, based on measurements and proportions, allows the identification of facial morphological characteristics, including the relative dimensions of the horizontal and vertical planes of the face. Researchers^(36)^ have stated that deviations from these proportions may indicate facial dimorphisms or specific clinical conditions requiring professional intervention. It can therefore be inferred that this type of analysis allows specialist to reflect on therapeutic limitations that negatively affect stomatognathic functions. Furthermore, asymmetries, according to the aforementioned authors^(36)^, may affect aesthetic perception, occlusion, facial growth, among others aspects. Thus, when numerical facial analysis was performed, the convergences and divergences among the three facial anthropometric methods employed in this study were subsequently verified. The results indicated that, regardless of the method, the proportions did not differ significantly when the facial thirds were the focus of the analysis, although this was not the case for hemifacial proportions. This aspect should therefore be carefully considered by professionals, particularly in speech-language pathology expert practice. As there are no comparable studies employing the same methods, it was not possible to contextualize the present findings within the existing literature.

However, it is important to emphasize that digital facial studies may not always be reliable. For this reason, it is recommended that multiple distance projections and image angles in both frontal and lateral views be employed, as well as the use of combined methods, such as photoanthropometry associated with morphological analysis^(4)^, to strengthen professional judgment.

Thus, it can be stated that, in both clinical and criminal investigations, it is crucial that analytical methods remain identical to ensure the consistency and reproducibility of results. Confidence in the scientific method employed is intended to guarantee that the legal system utilizes forensic information accurately and validly, underscoring that such practice must also be substantiated by scientific evidence.

In the present study, it was verified that measurements and proportions were greater in male participants when compared with female participants, revealing facial sexual dimorphism, as expected and already corroborated by the literature. In the present sample, the differences identified through calipers measurements and the application of Student's t-test were observed in the Al-Al and Ex-Ch proportions (both right and left). These findings are explained by the results obtained in research on dry skulls, specifically at the infraorbital foramen (right and left) and prosthion landmarks, which evidenced the aforementioned dimorphism in a Brazilian sample^(37)^.

The limitations of this study included the reduced number of anthropometric landmarks analyzed and the use of a method that does not correct for pixel size. This limitation was intentional, aiming to verify whether the proposed method would be robust for speech-language pathology practices involving facial anthropometry. The discrepancies observed among the methods lead to the conclusion that direct measurements should be prioritized whenever possible, and that, in forensic practice, both the expert and the technical assistant must employ the same method (including the analysis software) when comparing standard and questioned faces. It can be hypothesized that photographs may introduce distortions that do not necessarily reflect reality.

Further research involving facial anthropometry with SAPO® software is suggested to confirm or refute its accuracy for speech-language pathology purposes, as well as to compare the measurements obtained using different câmera brands within closed-circuit television (CCTV) systems, in order to validate or invalidate the application of photoanthropometry in forensic practice.

## CONCLUSION

Based on the results obtained with the studied sample, it can be concluded that:

- The direct measurements performed with the caliper were significantly different from those obtained by photoanthropometry, with higher values recorded by the direct method.
- Photoanthropometric measurements may vary depending on the software used, even if the same techniques for obtaining, calibrating, and measuring are applied (greater fragility of analysis: middle third of the face, regardless of sex).

Additionally, direct anthropometric measurements confirmed facial sexual dimorphism, as males presented higher values than females, with the exception of the middle third of the face.

## References

[1] Goodacre CJ, Roberts WE, Goldstein G, Wiens JP (2021). Does the stomatognathic system adapt to changes in occlusion? Best evidence consensus statement. J Prosthodont.

[2] Ujvári Z, Metzger M, Gárdonyi G (2023). A consistent methodology for forensic photogrammetry scanning of human remains using a single handheld DSLR camera. Forensic Sci Res.

[3] Cascos R, Ortiz del Amo L, Álvarez-Guzmán F, Antonaya-Martín JL, Celemín-Viñuela A, Gómez-Costa D (2023). Accuracy between 2D photography and dual-structured light 3D facial scanner for facial anthropometry: a clinical study. J Clin Med.

[4] César CPHAR, Bommarito S, Loyola RC, Lopes L, Machado APL, Azoni CAS, Benatti JF, Santos RS, Ribeiro VV (2024). Tratado de fonoaudiologia..

[5] Batista QR (2021). Possibilidades da fonoaudiologia forense na identificação humana. Rev Disc Uniflu..

[6] Reis WS (2018). Análise forense de imagens digitais no estudo de caso da ficha criminal de Dilma Rousseff. Análise..

[7] Souza PJS, Corrêa CC, Yaedú RYF, Berretin-Felix G (2021). Use of simulation technology for teaching of facial analysis in speech-language therapy. Online J Distance Educ Elearn.

[8] Grother P, Ngan M, Hanaoka K (2019). Face recognition vendor test (FRVT) Part 3: demographic effects.

[9] Nunes EL, Cardoso MCAF (2023). Validação de conteúdo de um instrumento de triagem em motricidade orofacial. Res Soc Dev..

[10] Bacci N, Briers N, Steyn M (2024). Prioritising quality: investigating the influence of image quality on forensic facial comparison. Int J Legal Med.

[11] Freepik (2025). Freepik.

[12] Blasi A, Nucera R, Rosinvalle V, Candida E, Grippaudo C (2022). Asymmetry index for the photogrammetric assessment of facial asymmetry. Am J Orthod Dentofacial Orthop.

[13] Aynechi N, Larson BE, Leon-Salazar V, Beiraghi S (2011). Accuracy and precision of a 3D anthropometric facial analysis with and without landmark labeling before image acquisition. Angle Orthod.

[14] Serrano LE (2024). Ferramenta digital para análise facial frontal na Odontologia utilizando a inteligência artificial.

[15] Stukaite-Ruibiene E, Ritz-Timme S, Cattaneo C, Obertova Z, Simkunaite-Rizgeliene R, Barkus A (2024). Photoanthropometric study: are non-professional photographs suitable for objective and reliable analysis of facial features?. Ann Hum Biol.

[16] Marchesan IQ, Berretin-Félix G, Genaro KF (2012). MBGR protocol of orofacial myofunctional evaluation with scores. Int J Orofacial Myology.

[17] Altman DG, Bland JM (1983). Measurement in medicine: the analysis of method comparison studies. Statistician.

[18] Alexandre NMC, Gallasch CH, Lima MHM, Rodrigues RCM (2013). A confiabilidade no desenvolvimento e avaliação de instrumentos de medida na área da saúde. Rev Eletr Enferm..

[19] Miola AC, Miot HA (2022). Comparing categorical variables in clinical and experimental studies. J Vasc Bras.

[20] Alves YB, Falcão TN, Fernandes LM, Nóbega JBM, Lima LNC, Machado CEP (2021). A fotoantropometria como método de análise facial para estimativa de idade forense: revisão sistemática. Res Soc Dev..

[21] Villanueva-Bonilla S, Saavedra-Layera L, Vergara-Núñez C (2018). Comparación de mediciones antropométricas directa y con sistema de imagen 3D, en adultos jóvenes. Rev Clin Periodoncia Implantol Rehabil Oral.

[22] Smith K, Wilkinson C (2024). The doppelgänger effect? A comparative study of forensic facial depiction methods. Forensic Sci Int.

[23] Duckett LJ (2021). Quantitative research excellence: study design and reliable and valid measurement of variables. J Hum Lact.

[24] Ferreira EAG, Duarte M, Maldonado EP, Burke TN, Marques AP (2010). Postural assessment software (PAS/SAPO): validation and reliability. Clinics (Sao Paulo).

[25] Miot HA, Pivotto DR, Jorge EN, Mazeto GMFS (2008). Avaliação de parâmetros métricos oculares pela fotografia digital da face: uso do diâmetro da íris como unidade de referência. Arq Bras Oftalmol.

[26] Stocchero IN, Stocchero GF, Stocchero GF, Fonseca ASF (2012). Método de avaliação da suspensão do SMAS no rejuvenescimento facial. Rev Bras Cir Plást.

[27] Seneschal J, Duplaine A, Maillard H, Passeron T, Andreu N, Lassalle R (2021). Efficacy and safety of tacrolimus 0.1% for the treatment of facial vitiligo: a multicenter randomized, double-blinded, vehicle-controlled study. J Invest Dermatol.

[28] Sezgin N, Karadayi B (2023). Sex estimation from biometric face photos for forensic purposes. Med Sci Law.

[29] Farkas LG, Tompson B, Phillips JH, Katic MJ, Cornfoot ML (1999). Comparison of anthropometric and cephalometric measurements of the adult face. J Craniofac Surg.

[30] Bolzan GP, Costa MC, Correa EC, Haeffner LS (2014). Agreement between anthropometry and cephalometry methods in classification of the facial type. Rev CEFAC.

[31] Choi KY (2015). Analysis of facial asymmetry. Arch Craniofac Surg.

[32] Nakamura T, Okamoto K, Maruyama T (2001). Facial asymmetry in patients with cervicobrachial pain and headache. J Oral Rehabil.

[33] Schindelin J, Arganda-Carreras I, Fernández M, Kaynig AK, Pietzsch PC, Preibisch S (2015). The IMAGEJ® ecosystem: an open platform for biomedical image analysis. Mol Reprod Dev.

[34] Falcão TN, Silva MRS, Lima FS, Souza RF, Pereira JM, Almeida AC (2021). Photoanthropometry in forensics: comparison of facial images with frontal and lateral views. Res Soc Dev..

[35] Jodeh DS, Rottgers SA (2019). High-fidelity anthropometric facial measurements can be obtained from a single stereophotograph from the Vectra H1 3-dimensional camera. Cleft Palate Craniofac J.

[36] Pinto PHV, Leal RM, Silva RF, Gamba TO, Leite AF, Paranhos LR (2021). Comparative analysis between linear measures from bidimensional and three-dimensional images of the face for human identification purpose: a pilot study. J Orofac Sci.

[37] Andrade NN, Mathai P, Aggarwal N, Bonanthaya K, Paneerselvam E, Manuel S, Kumar VV, Rai A (2021). Oral and maxillofacial surgery for the clinician..

[38] Almeida EC, Silva FR, Pereira JA, Lima TS, Costa AR (2010). Investigação do sexo através de uma área triangular facial formada pela interseção dos pontos: forame infraorbital direito, esquerdo e o próstio, em crânios secos de adultos. Rev Ciênc Méd Biol.

