# Supplementary material for: Reliability between measurements obtained by caliper and digital photography in facial anthropometry
Source: Codas. 2026 Mar 31;38(3):e20250193. doi: 10.1590/2317-1782/e20250193en (PMC13142683; doi:10.1590/2317-1782/e20250193en)
Supplement: Translation

# Translation: Confiabilidade entre as medidas obtidas pelo paquímetro e por fotografia digital na antropometria facial

**DOI:** 10.1590/2317-1782/e20250193pt

**Authors:** Rayssa Silva Santos Andrade, Paulo Vitor de Oliveira Santos Matos, Maria Inês Beltrati Cornacchioni Rehder, Raphaela Barroso Guedes-Granzotti, Kelly da Silva, Carla Patrícia Hernandez Alves Ribeiro César

**Affiliations:** 1 Departamento de Fonoaudiologia, Universidade Federal de Sergipe – UFS - São Cristóvão (SE), Brasil.; 2 Pós-graduação em Perícia Fonoaudiológica, CEFAC – Saúde Educação - Rio Claro (SP), Brasil.; 3 Parole Fonoaudiologia Clínico Científica - Rio Claro (SP), Brasil.; 4 Departamento de Fonoaudiologia, Universidade Federal de Sergipe – UFS - Lagarto (SE), Brasil.

**Keywords:** Antropometria, Face, Fonoaudiologia, Ciências Forenses, Metodologia

## Abstract

**Objetivo:**

Investigar a confiabilidade entre medidas diretas e indiretas na antropometria facial.

**Método:**

Amostra constituída por 60 participantes, com idades entre 16 e 48 anos. Após a aplicação dos critérios de elegibilidade, foram procedidos exames faciais antropométricos objetivos indiretos (softwares SAPO® e IMAGEJ®) e diretos em norma frontal, por meio do uso do paquímetro digital nos pontos antropométricos: glabela, subnasal, gnátio, alar, exocanto e cheilion com os pacientes sentados, em posição confortável e cabeça em posição natural. Dois examinadores treinados e calibrados realizaram as medições por três vezes para a obtenção de média aritmética. Os participantes foram fotografados para a realização das medições indiretas. Os resultados foram analisados por estatística descritiva (medidas de frequência absoluta e relativa e medidas sínteses de tendência central e dispersão) e inferencial (teste Chi-quadrado, Bland-Altman, t pareado e Coeficiente de Correlação Intraclasse) considerando-se significância de 5%.

**Resultados:**

Houve concordância intra e interobservadores, dimorfismo sexual (medidas femininas menores), valores maiores na antropometria direta quando comparados com a fotoantropometria, diferenças entre os softwares utilizados (em terço médio para ambos os sexos e terço inferior para o masculino) e na análise facial numérica, discordância na análise das hemiface, independente da técnica utilizada.

**Conclusão:**

Houve divergência entre as técnicas utilizadas, devendo o profissional levar esse aspecto em consideração. Comparações antropométricas faciais devem ser efetivadas pelo uso de um mesmo software a fim de evitar divergências de análises.

## INTRODUÇÃO

Em Motricidade Orofacial, mensurar estruturas orofaciais possibilita entender a contribuição da forma e suas relações com as funções e vice-versa^(1)^. Com o propósito pericial, a documentação fotográfica auxilia na identificação humana e na reconstrução forense, sendo considerada padrão-ouro por possibilitar a coleta de informações adicionais sempre que forem necessárias, principalmente na revisão de casos criminais, sendo considerada uma medida simples, barata e precisa^(2)^. Assim, pode ser realizada com uma única câmera fotográfica, fornecendo imagens em segunda dimensão (2D) e com várias câmeras ou por escaneamento facial, possibilitando imagens tridimensionais (3D)^(3)^.

Desta forma, a antropometria facial permite, de maneira objetiva e com pontos de referência anatômicos, aferir e comparar medidas entre pessoas^(4)^. Para a avaliação direta pode ser utilizado o paquímetro e para a indireta, a fotografia, cujas medidas podem ser obtidas através de *softwares*. O *software* para avaliação postural (SAPO®), de acesso livre, pode ser utilizado para a aferição indireta de medidas da face em perícias fonoaudiológicas^(5)^. Não obstante, outro programa gratuito que pode ser utilizado nas perícias, o IMAGEJ®, é uma ferramenta digital que permite escalabilidade, uma vez que com o uso de *plug-ins* é possível acrescentar algoritmos para a análise de imagens^(6)^. No entanto, a capacitação faz-se premente, uma vez que os resultados das medidas obtidas dependem da *expertise* do usuário/clínico^(7)^.

Cabe ressaltar, no entanto, que em relação ao uso de *softwares*, a literatura infere que, apesar da análise facial computadorizada oferecer maiores recursos de análises e precisão, pode haver erros como falsos-positivos ou falsos-negativos^(8)^. É visível, portanto, que devido aos poucos estudos que relacionem a confiabilidade das medições diretas (com o uso do paquímetro diretamente no tecido cutâneo facial do paciente) e indiretas, com os diversos *softwares* na antropometria facial dentro da Fonoaudiologia, há a necessidade de verificação da possibilidade quanto ao seu uso de forma padronizada e com bases científicas que subsidiem o seu uso profissional.

Desse modo, este estudo tem como objetivo investigar a confiabilidade entre as medidas diretas e as indiretas na antropometria facial.

## MÉTODO

Estudo transversal, descritivo e observacional, foi devidamente aprovado pelo Comitê de Ética em Pesquisa da Universidade Federal de Sergipe (CAAE nº 78821524.9.0000.5546 e Processo nº 6.823.063), sendo que os participantes assinaram o Termo de Consentimento Livre e Esclarecido.

A amostra foi configurada por conveniência e composta por 60 participantes, com idades entre 16 e 48 anos (média: 23,98, DP: 7,47), sendo 30 (50%) do gênero masculino e 30 (50%) do feminino, todos considerados hígidos. Salienta-se que uma adolescente de dezesseis anos foi incluída, no intuito de finalizar a composição da amostra, uma vez que a quantidade de participantes se deu pela realização do cálculo amostral por meio do Coeficiente de Correlação Intraclasse (ICC), sendo o mínimo aceitável de 0,75 e um ICC esperado de 0,90, o nível de significância de 5% (α = 0,05) e o poder estatístico de 80% (1–β = 0,80), obtendo-se o valor supracitado. Para evitar viés de confusão sobre a interferência dessa participante no estudo, mesmo excluindo-a da amostra, o ICC não se modificou, o que permitiu que mantivéssemos essa participante no estudo.

Os critérios de inclusão foram: idade, oclusão dentária harmoniosa (relação de primeiro molar em degrau mesial e relação maxilomandibular equilibrada), modo respiratório nasal (pela possibilidade de uso nasal exclusivo por mais de dois minutos aferido com cronômetro) e pontuação na triagem em motricidade orofacial com escores (TMO) de até um ponto^(9)^. Os critérios de exclusão foram: alterações oclusais (Classes II e III de Angle, mordida aberta, mordida cruzada e sobremordida), modo respiratório oral (pela queixa ou tratamentos realizados), histórico positivo para desordens neurológicas que afetam a mobilidade e/ou tônus muscular, alterações motoras que pudessem interferir na estabilidade corpórea e, portanto, na estabilidade durante os procedimentos, em tratamento (ortodôntico e/ou fonoaudiológico), cirurgias plásticas e outras alterações estruturais no complexo crânio-oro-cervical de diferentes ordens (hereditárias, genéticas ou ambientais, como traumas e queimaduras faciais).

Imagens que não atendessem aos critérios de boa ou ótima qualidade, segundo a classificação adotada^(10)^, foram refeitas ou excluídas na impossibilidade de outra tomada.

Para a aplicação dos critérios de elegibilidade, os participantes foram triados pelo TMO^(9)^, sendo que na verificação de pontuação igual ou superior a dois pontos, os participantes foram encaminhados para avaliação completa na área e excluídos da pesquisa. A seguir, foram realizados os exames faciais antropométricos objetivos, com o uso do paquímetro digital marca Digital Caliper e de dois *softwares*: o SAPO® e o IMAGEJ®.

Os procedimentos foram realizados em ambiente silencioso e privado, em horário pré-determinado e em comum acordo com o participante. Na ocasião, foram obtidas informações sobre a identificação, os dados socioeconômicos e aplicados os procedimentos da pesquisa, descritos a seguir.

Para a avaliação objetiva direta, foi utilizado paquímetro digital e foram delineados na face (previamente higienizada com álcool) com o auxílio de um lápis dermatográfico. As medições foram calculadas com precisão de 0,01 mm aplicando-se a menor pressão possível na superfície da pele dos participantes. Nove pontos antropométricos foram demarcados: Glabela (G), Subnasal (Sn), Gnátio (Gn), Canto Externo do Olho (Ex), Asas do Nariz (Ald-Ale), a fim de ser possível a obtenção dos terços faciais médio (G-Sn) e inferior (Sn-Gn), assim como a distância entre os cantos externos do olho direito à comissura direita do lábio (ExD-ChD) e dos cantos externos do olho esquerdo à comissura esquerda do lábio (ExE-ChE), conforme Figura 1.

**Figura 1:** Pontos antropométricos utilizados na presente pesquisa, tanto na antropometria direta quanto na indireta

**Legenda:** Glabela (G), Subnasal (Sn), Gnátio (Gn), Canto Externo do Olho (Ex), Asas do Nariz (Ald-Ale). Fonte: Freepik^(11)^

*Observação*: o traço horizontal vermelho apresenta 10 mm e foi introduzido para a parametrização das aferições nas fotoantropometrias realizadas

As medidas foram tomadas por três vezes, por dois avaliadores, de forma independente, a fim de serem obtidas as médias aritméticas. Faz-se importante mencionar que os avaliadores foram previamente capacitados para essa finalidade, sendo que a capacitação foi procedida com curso teórico-prático de 14 horas, contendo, ao final, avaliação com a aferição facial tanto pela antropometria direta e pela indireta, comparando-se os resultados obtidos com uma das autoras que foi considerada padrão-ouro na área pela atuação profissional em perícia, em motricidade orofacial, por ser docente da área e apresentar doutorado. Somente após essa etapa, denominada de calibração dos avaliadores, deu-se início à antropometria.

Para as tomadas fotográficas e antropometria direta, todos os participantes permaneceram sentados em uma cadeira sem apoio para a cabeça, com os pés no chão, sem óculos e maquiagem, cabelo preso, com cabeça posicionada no plano horizontal de Frankfurt, oclusão dentária respeitando o espaço funcional livre (sem contato entre as cúspides oclusais) e com fundo branco. Houve a orientação de que os participantes mantivessem a face neutra (sem o esboço de expressões faciais nem contrações). A iluminação do ambiente foi considerada satisfatória para as tomadas fotográficas. Os resultados foram anotados em folha própria para tal finalidade. Cabe ressaltar que foi considerada assimetria entre as hemifaces com valores iguais ou superiores a 2 mm^(12,13)^, em todos os métodos de aferição.

A seguir, foram feitos os registros fotográficos (fotografia 2D) do participante já com os pontos antropométricos demarcados na pele. Foi utilizado o acionamento do modo câmera fotográfica de aparelho celular android Samsung Galaxy S23®, no modo “rosto” (nessa modalidade há o enquadramento dentro de um quadrado branco para aprimorar a padronização do posicionamento das faces), uma vez que literatura^(14)^ evidenciou que ferramentas digitais inclusas em celulares permitem capturas de imagens com boa qualidade para a medição da face e, portanto, para o uso clínico e que o uso de equipamentos não profissionais podem ser utilizados na fotoantropometria^(15)^. O aparelho foi fixado em um tripé, disponibilizado a 1,5 m de distância do participante e a tomada fotográfica foi procedida na norma frontal. Em seguida, as imagens foram salvas no computador para serem abertas em dois *softwares* para análise: o SAPO® e o IMAGEJ®, a fim de medir os mesmos pontos faciais e verificar se as proporções das medidas obtidas com o uso do paquímetro seriam ou não mantidas com o uso dos *softwares* eleitos para tal finalidade.

Para tanto, as imagens foram inseridas no *software* Powerpoint da Microsoft®, e ajustadas em tamanho e rotação e, a seguir, foram introduzidos linhas (horizontal e vertical) de 10mm (acionando-se o ícone de desenho do próprio *software* e conferindo a medida pela janela de medição que se abre durante o desenho da linha), sendo estas introduzidas nas imagens a fim de executar a padronização das medidas em ambos *os softwares* utilizados, tanto na posição horizontal quanto na vertical. As mesmas medidas foram tomadas por três vezes, obtendo-se a média aritmética, sendo os resultados transpostos para folha de registros. A fim de verificar a concordância intraobservador, após duas semanas da obtenção das primeiras medidas na fotoantropometria (a fim de evitar viés de memória: *recall bias* e aliviar a fadiga das aferições já realizadas, garantindo a confiabilidade intraexaminador) em cada *software* testado, de forma randomizada, doze fotografias foram novamente medidas. Caso houvesse concordância intra e interobservador, foi dado prosseguimento à coleta de dados.

Com os valores obtidos a partir dos pontos antropométricos utilizados, foi realizada a análise facial numérica dos terços da face e das hemifaces, assinalando-se, por meio dessa análise, se eram maiores, menores ou iguais, registrando-se o de maior lado (no caso das hemifaces) e o maior terço facial, conforme protocolo MBGR^(16)^.

As mesmas medidas foram tomadas por três vezes, obtendo-se a média aritmética, sendo os resultados transpostos para folha de registros. Desta forma, ao final da coleta, os dados foram organizados em planilhas no Microsoft Office Excel 2013 (Microsoft® Corp., Redmond, WA, USA). Foi verificada a normalidade dos dados a partir do teste de Shapiro-Wilks. A análise dos resultados foi realizada por meio de estatística descritiva (frequências, médias e desvio-padrão) e a técnica de concordância Bland-Altman^(17)^.

A concordância intraobservador foi realizada pelo teste t pareado e interexaminador pelo Coeficiente de Correlação Intraclasse (ICC) por meio da análise de doze imagens selecionadas por randomização (feita pelo Excel do pacote Microsoft®) 30 dias após as medidas iniciais, com 20% da amostra (12 novas aferições para cada avaliador). Cabe ressaltar que tais análises foram realizadas apenas nas antropometrias indiretas pelas dificuldades de refazer as medições presencialmente (necessárias para a antropometria direta). A análise facial numérica foi submetida à análise estatística pelo teste de Igualdade de Proporções. O *software* utilizado para todas as análises foi o JAMOVI. Foi adotado nível de significância quando o p-valor foi menor do que 5%.

## RESULTADOS

Para verificar a concordância intraobservador (teste t pareado e o ICC), as medidas aferidas pelo avaliador 1, após 30 dias, em 20% da amostra revelaram não haver diferenças estatisticamente significativas pelo uso dos métodos direto e indiretos, como pode ser observado na Tabela 1.

**Tabela 1:** Análises estatísticas (testes t pareado e ICC) da concordância intraobservadores nos dois *softwares* utilizados

Avaliador	Software	Teste Est.	Terço Médio	Terço Inferior	AlarD - AlarE	Hemiface D	Hemiface E
**Avaliador 1**	SAPO®	T pareado	0,128	0,286	0,554	0,075	0,610
		ICC	0,999	0,997	0,999	0,998	0,999
	IMAGEJ®	T pareado	0,924	0,976	0,886	0,820	0,292
		ICC	1,000	0,999	0,999	0,999	1,000
**Avaliador 2**	SAPO®	T pareado	0,323	0,970	0,632	0,799	0,548
		ICC	0,993	0,995	0,990	0,996	0,995
	IMAGEJ®	T pareado	0,008	0,345	0,838	0,722	0,439
		ICC	0,997	0,999	0,996	0,999	0,999

**Legenda:** D=direita, E=esquerda, ICC=Coeficiente de Correlação Intraclasse, Teste Est.= Teste Estatístico. **Fonte:** autores.

A análise da concordância interobservadores pelo teste ICC realizada pelas médias obtidas dos pontos antropométricos utilizados revelou que as medidas tomadas pelo avaliador 1 e refeitas pelo avaliador 2 foram concordantes entre si, tanto pelo *software* SAPO quanto pelo IMAGEJ, conforme Tabela 2. Considerando que o ICC foi superior a 0,9, indicando excelente concordância e que houve consistência nas medições realizadas pelos avaliadores.

**Tabela 2:** Análise da concordância interobservadores entre o avaliador 1 e o avaliador 2, nos dois *softwares* utilizados

ICC	Softwares	TM A1	TM A2	TI A1	TI A2	Al-Al A1	Al-Al A2	HFD A1	HFD A2	HFE A1	HFE A2
	**SAPO®**	0,981		0,987		0,980		0,983		0,976	
	**ImageJ®**	0,992		0,999		0,986		0,996		0,993	

**Legenda:** A1=avaliador 1, A2=avaliador 2, Al=alar, D=direita, E=esquerda, HF=hemiface (medida de exocanto a cheilion), TI=terço inferior, TM=terço médio, Teste estatístico utilizado: Coeficiente de Correlação Intraclasse (ICC). **Fonte:** autores

Os resultados comparativos das médias obtidas pelos métodos utilizados evidenciaram que as medições diretas foram diferentes e maiores aos obtidos pela fotoantropometria 2D, sendo o método direto considerado diferente das fotoantropometrias de acordo com o teste estatístico utilizado, o Bland-Altman. Quando comparadas as medidas das fotoantropometrias dos *softwares* utilizados, a única diferença, para o sexo feminino, foi a do terço médio da face, enquanto para o masculino, os terços da face (tanto o médio quanto o inferior). Dessa forma, a fotoantropometria obtida pelo SAPO® e pelo IMAGEJ® são parcialmente similares, a depender do ponto antropométrico analisado e do sexo do participante (Tabela 3).

**Tabela 3:** Medições obtidas pelo uso direto do paquímetro digital (avaliação direta) e com o uso dos *softwares* (SAPO® e IMAGEJ®)

Sexo/Resultados		Asa do Nariz			Hemiface D			Hemiface E			Terço Médio			Terço Inferior		
		Paq	SAPO®	IMAGEJ®	Paq	SAPO®	IMAGEJ®	Paq	SAPO®	IMAGEJ®	Paq	SAPO®	IMAGEJ®	Paq	SAPO®	IMAGEJ®
Fem.	Média	33,5	23,3	23,7	69,3	41,0	41,4	68,5	40,9	41,3	56,9	38,5	39,2	59,7	37,4	37,6
	Mediana	33,5	23,5	23,4	70,2	40,6	41,0	68,3	40,7	41,3	54,3	38,5	38,3	60,5	37,8	37,7
	DP	3,80	2,54	2,80	3,86	4,96	5,21	3,92	4,68	5,11	5,72	6,23	7,09	6,74	4,11	4,18
	Mínimo	26,2	18,5	18,0	60,1	31,0	30,4	58,7	30,8	30,1	46,9	27,4	26,8	47,4	28,7	28,9
	Máximo	39,8	29,5	30,5	76,2	49,3	50,8	75,1	49,6	51,7	68,5	52,2	52,8	70,2	44,5	44,3
	Bland-Altman	Paq e SAPO®: t = -13, df = 29, p-value = 2e-13: Paq e IMAGEJ®: t = 12, df = 26, p-value = 2e-12: SAPO® e IMAGEJ®: t = -1, df = 26, p-value = 0,3,			Paq e SAPO®: t = 29, df = 29, p-value = <2e-16; Paq e IMAGEJ®: t = 29, df = 29, p-value = <2e-16; SAPO® e IMAGEJ®: t = -1,7, df = 29, p-value = 0,1			Paq e SAPO®: t = 29, df = 29, p-value = <2e-16; Paq e IMAGEJ®: t = 28, df = 29, p-value = <2e-16; SAPO® e IMAGEJ®: t = -2, df = 29, p-value = 0,06			Paq e SAPO®: t = 16, df = 29, p-value = 7e-16; Paq e IMAGEJ®: t = 14, df = 29, p-value = 1e-14; SAPO® e IMAGEJ®: t = -0,28, df = 29, p-value = 0,01			Paq e SAPO®: t = 22, df = 29, p-value = <2e-16; Paq e IMAGEJ®: t = 24, df = 29, p-value = <2e-16; SAPO® e IMAGEJ®: t = -0,83, df = 29, p-value = 0,4		
Masc.	Média	36,9	24,5	24,6	72,9	41,0	40,8	72,1	40,8	41,0	56,9	36,5	37,1	64,8	41,8	37,1
	Mediana	37,2	24,9	24,9	73,7	41,8	41,6	72,3	41,6	41,2	57,5	37,0	37,1	66,4	41,2	37,1
	DP	3,21	2,82	3,18	4,61	4,59	5,72	3,95	4,63	4,99	5,28	6,39	7,28	7,30	4,85	7,28
	Mínimo	30,7	19,4	19,2	62,4	29,4	24,2	64,9	28,6	27,9	45,6	25,7	25,3	37,0	31,4	25,3
	Máximo	43,0	29,8	31,1	79,6	49,2	49,0	77,8	47,9	50,2	71,5	49,1	49,5	77,0	52,3	49,5
	Bland-Altman	Paquímetro e SAPO®: t = 15, df = 29, p-value = 3e-15; Paquímetro e IMAGEJ®: t = 13, df = 29, p-value = 7e-14; SAPO® e IMAGEJ®: t = -0,69, df = 29, p-value = 0,5			Paquímetro e SAPO®: t = 35, df = 29, p-value = <2e-16; Paquímetro e IMAGEJ®: t = 22, df = 29, p-value = <2e-16; SAPO® e IMAGEJ®: t = 0,15, df = 29, p-value 0,9			Paquímetro e SAPO®: t = 33, df = 29, p-value = <2e-16; Paquímetro e IMAGEJ®: t = 30, df = 29, p-value = <2e-16; SAPO® e IMAGEJ®: t = -0,96, df = 29, p-value = 0,3			Paquímetro e SAPO®: t = 17, df = 29, p-value = <2e-16; Paquímetro e IMAGEJ®: t = 14, df = 29, p-value = 8e-15; SAPO® e IMAGEJ®: t = -2,2, df = 29, p-value = 0,03			Paquímetro e SAPO®: t = 19, df = 29, p-value = <2e-16; Paquímetro e IMAGEJ®: t = 18, df = 29, p-value = <2e-14; SAPO® e IMAGEJ®: t = 4,4, df = 29, p-value = 1e-0,4		

**Legenda:** df=graus de liberdade, Paq=paquímetro, t=tendência, Estatística Bland-Altman^(15)^. **Fonte:** autores

Na Tabela 4 pode-se observar as convergências das análises faciais numéricas entre os terços da face na avaliação direta e indireta que não revelou diferença significância pelo teste estatístico utilizado, embora quando as proporções das hemifaces foram comparadas, houve significância, mostrando divergências em todos os métodos utilizados na presente pesquisa.

**Tabela 4:** Análise facial numérica comparada entre o paquímetro digital e as aferições fotoantropométricas (SAPO® e IMAGEJ®)

Variável de Análise	Método	Classificação			Total	P-valor
		Nº de participantes com Terço Inf. >	Nº de participantes com Terço Inf. <	Nº de participantes com Terços Semelhantes		
**Terços da Face**	IMAGEJ®	30	20	10	60	0,254
	Paquímetro	42	12	6	60	
	SAPO®	34	16	10	60	
	**Total**	106	48	26	180	
		Nº de participantes com Hemiface D >	Nº de participantes com Hemiface D <	Nº de participantes com Hemifaces Semelhantes	Total	P-valor
**Hemiface**	IMAGEJ®	05	02	53	60	<0,001
	Paquímetro	20	10	30	60	
	SAPO®	05	01	54	60	
	**Total**	30	13	137	180	

**Legenda:** D=direita; Inf.=inferior, Nº=número, >=maior, <=menor. Teste estatístico utilizado: Teste Chi-Quadrado. **Fonte:** autores

Na Tabela 5, pela aferição realizada com o paquímetro, foi possível constatar que houve dimorfismo sexual das médias obtidas a partir dos pontos antropométricos alD-alE, Sn-Gn, ExD-ChD e ExE-ChE, com exceção do terço médio da face. De forma geral, as medidas faciais femininas foram menores do que as masculinas, pelo teste t de Student.

**Tabela 5:** Comparação das médias obtidas com o paquímetro (avaliação antropométrica direta) entre os sexos masculino e feminino

Resultados/ Proporções	Asa do Nariz		Hemiface D		Hemiface E		Terço Médio		Terço Inferior	
	Masc.	Fem.	Masc.	Fem.	Masc.	Fem.	Masc.	Fem.	Masc.	Fem.
**Média**	36,9	33,5	72,9	69,3	72,1	68,5	56,9	56,9	64,8	59,7
**Mediana**	37,2	33,5	73,7	70,2	72,3	68,3	57,5	54,3	66,4	60,5
**DP**	3,21	3,8	4,61	3,86	3,95	3,92	5,28	5,72	7,30	6,74
**p-Valor**	<0,001		0,002		0,001		1,0		0,007	

**Legenda:** Fem.=feminino, Masc.=masculino. Teste estatístico utilizado: teste t de Student. **Fonte:** autores

## DISCUSSÃO

O objetivo da presente pesquisa foi investigar a confiabilidade entre medidas diretas e indiretas na antropometria facial em adultos brasileiros, no intuito de comparar os critérios metodológicos utilizados tanto na perícia da face quanto na prática clínica fonoaudiológica. Dessa forma, espera-se oferecer evidências científicas que possam subsidiar a escolha profissional por um método em detrimento a outros. Pesquisadores^(18)^ afirmaram que pesquisas e estudos na área de saúde devem estabelecer e documentar a validade, bem como verificar a confiabilidade dos instrumentos de medida utilizados na prática profissional. Ratificando o exposto, investigadores^(19)^ complementaram que comparar variáveis categóricas é uma demanda frequente em estudos da saúde e que pode resultar em diferentes conclusões inferenciais conforme o método de análise aplicado, reforçando que a escolha da técnica de análise exige fundamentação teórica e que os parâmetros de uso devem ser descritos.

Em relação ao tema deste estudo, a antropometria facial pode ser realizada pelo emprego de diferentes recursos, sendo que a usada em fotografias (denominada por fotoantropometria) tem algumas vantagens como o fato de não ser invasiva, ter baixo custo, ser rápida, reprodutível, além de as imagens poderem ser arquivadas para futuras comparações de resultados^(20)^. No entanto, a literatura apresenta também desvantagens. A primeira, segundo a literatura^(21)^, é que por ser uma representação bidimensional de uma realidade tridimensional, pode limitar a abordagem profissional. Outra é a de que, nas exportações das imagens detalhes cruciais podem ser perdidos^(20)^. Há ainda a possibilidade da presença de artefatos de imagem, de deformações (posturais ou do próprio equipamento) que podem reduzir a precisão morfológica, diminuindo a credibilidade da análise^(22)^.

Outro aspecto a ser salientado é a possibilidade de que a presença de expressões faciais emocionais possa influenciar significativamente o processo de comparação de imagens faciais. Frente ao exposto, uma das medidas adotadas na presente pesquisa foi a de solicitar aos participantes que permanecessem com a face a mais neutra possível, evitando medidas distorcidas tanto na avaliação direta quanto na indireta. A medida adotada de reforçar os pontos já demarcados na face nas fotografias tentou garantir que os mesmos pontos fossem medidos, uma vez que ligeiras alterações para mais ou para menos poderiam, por si só, gerar medidas distintas entre os métodos do estudo.

Diz-se de um procedimento confiável aquele que obtém sempre o mesmo resultado, dentro das variações aceitas, em medidas repetidas de uma mesma variável^(23)^. Dessa maneira, segundo um estudo^(24)^, o *software* de avaliação postural (SAPO®) foi descrito como uma ferramenta confiável para análise postural porque a concordância entre os avaliadores e os interavaliadores foi muito boa ou excelente. Além disso, para o fator repetibilidade, os dados também sugeriram boa confiabilidade intra-avaliador. Não obstante, os autores consideraram a variável da experiência com o *software* como um fator determinante para uma maior confiabilidade. Cabe ressaltar que esse *software* é de uso comum na Fisioterapia, sem estudos publicados na Fonoaudiologia, justificando a realização da presente pesquisa. Já em relação ao IMAGEJ®, diferentes pesquisas têm utilizado o *software* na identificação facial humana, como um estudo sobre o uso do diâmetro da íris como unidade de referência na fotografia digital^(25)^, nas comparações de áreas faciais pré e pós-ritidoplastias^(26)^, para comparar áreas pré e pós-tratamento com pomada de tracolimo a 0,1% para vitiligo facial^(27)^ e na antropometria forense para a estimativa do sexo, por exemplo^(28)^.

Independentemente do método utilizado, as aferições realizadas por três vezes em cada referência antropométrica permitiram que houvesse concordância intra-examinador. Isso implica que ao ser adotado um método, a comparação pode ser empregada desde que respeitados os mesmos princípios/técnicas de realização, favorecendo a replicabilidade de dados. Mesmo assim, os valores obtidos na avaliação direta foram maiores do que os obtidos na fotoantropometria, pela técnica empregada, independente da variável sexo e ponto antropométrico estudado. Isso ocorreu pois o paquímetro permite dimensionar diretamente os pontos utilizados, podendo ser considerado como referência no estudo com tecidos moles. Além disso, estudo já evidenciou que a diferença entre os pontos cefalométricos e os medidos com o paquímetro no tecido mole variaram entre 1,2mm e 1,5mm, em média, para o terço inferior e superior, respectivamente, o que justifica a medição repetida a fim de evitar erros de análise^(29)^. Outra pesquisa^(30)^, que avaliou o tipo facial em crianças, encontrou ligeiras concordâncias entre o método cefalométrico e as medidas diretas com o paquímetro, mostrando que a aferição por meio do uso do paquímetro é uma alternativa viável para a prática clínica em motricidade orofacial.

Já os *softwares*, mesmo fazendo-se uma calibração prévia de dez milímetros (um centímetro) para padronizar os resultados entre os *softwares*, tem-se que as medidas obtidas são adimensionais, ou seja, em pixels. Para minimizar esse viés, na comparação entre medidas diretas e indiretas infere-se que outras técnicas necessitem ser aplicadas, como o uso de índices faciais (pela divisão de uma dimensão facial menor por outra dimensão maior e o resultado for multiplicado por 100. Exemplo: en-en/al-al), como sugerido por pesquisadores^(15)^. Cabe salientar que os índices não foram foco do presente estudo, sendo uma sugestão para estudos futuros.

Quando comparadas as medidas obtidas na face, tanto a direta quanto as indiretas (mensuradas com os *softwares* SAPO® e o IMAGEJ®), os terços da face foram os que apresentaram discrepâncias tanto entre os métodos quanto entre os sexos, sendo o terço médio para ambos os sexos e o terço inferior apenas para o sexo masculino. Estudo^(12)^ demonstrou que os pontos antropométricos localizados no terço inferior da face apresentam maior índice de assimetria que os demais, mesmo na avaliação com imagens em 3D, devendo esse aspecto ser levado em consideração. Os pesquisadores^(12)^ salientaram ainda que mesmo em faces consideradas assimétricas, algumas medidas não evidenciaram diferenças nas medidas aferidas. Assim, novos estudos precisam ser efetivados a fim de estabelecer valores fidedignos para análise e conduta clínica.

Pesquisador acrescentou que pequenas diferenças (entre 3-4mm para análises lineares e 3°-4° para as angulares) não são reconhecidas como assimetrias. Seriam tidas como assimetrias funcionais. Já para a análise funcional do sorriso, por exemplo, diferenças de 3mm são suficientes para que a assimetria seja visível e não percebida como esteticamente agradável, podendo ser uma assimetria patológica^(31)^. Em virtude do exposto, considera-se uma limitação do presente estudo, a não aplicação do índice de assimetria proposto pela literatura^(32)^.

No entanto, pode-se inferir que mesmo que haja diferenças estaticamente significativas, podem não revelar impactos na prática clínica, como por exemplo, diferenças menores do que 2,0 mm. Isso porque existe variabilidade na aferição intra e interavaliador, julgando-se mínimas tais diferenças, além da possibilidade da existência de variações anatômicas individuais. Cabe ressaltar que, na prática profissional, há a necessidade da calibração prévia (no caso do presente estudo: 10mm) para a fotoantropometria e a replicação do método da antropometria direta de aferição por três vezes a fim da obtenção de uma média, para que as mensurações sejam sistemáticas, replicáveis e, consequentemente, mais precisas.

Outro aspecto a ser salientado é que os *softwares* foram compatíveis entre si nos valores obtidos para o sexo feminino para Al-Al, ExD-ChD, ExE-ChE e Sn-Gn e para o sexo masculino em Al-Al, ExD-ChD e ExE-ChE, sendo que esses aspectos devem ser levados em consideração no uso de *softwares* tanto na prática clínica quanto na forense. Fatores como a reflectância da pele podem interferir na precisão de sistemas de reconhecimento facial, a depender da qualidade da aquisição da imagem, sendo que essa variável deve ser levada em consideração no momento das análises antropométricas indiretas.

Frente aos resultados obtidos, pode-se afirmar que, a depender dos pontos antropométricos de análise, diferentes *softwares* podem ser usados na fotoantropometria, sendo que as medidas das hemifaces e da asa do nariz, independentemente da variável sexo, podem ser confiáveis. Destaca-se o uso do SAPO® como um *software* que pode ser utilizado por fonoaudiólogos na antropometria facial, seja na prática clínica ou na comparação binária de faces da perícia fonoaudiológica. O mesmo pode-se afirmar para o IMAGEJ®^,^ que, de acordo com a literatura^(33)^, é utilizado em pesquisas pela sua versatilidade, acessibilidade e disponibilização em código aberto, sendo amplamente utilizado também em casos periciais. Já a Polícia Federal, por exemplo, utiliza o SAFF-2, de uso exclusivo (não disponibilizado como código aberto), com precisão nas análises fotoantropométricas na maioria das medidas utilizadas na pesquisa de comparação dos pontos antropométricos (de um total de 27 pontos, 21 foram concordantes) entre as normas frontal e lateral dos participantes^(34)^. Na atualidade, a escolha tem sido pela estereofotogrametria, que permite a visão em terceira dimensão de uma imagem em 2D reformatada, qualificando-a e tornando-a mais realista, segundo a literatura^(35)^. No entanto, apesar de suas vantagens, a tecnologia que permite a captura de imagens faciais em 3D, com mais de uma câmera fotográfica ou com escâner facial, em diferentes ângulos, para possibilitar a visão da face com sua profundidade, ainda não é difundida. Nesse contexto, as fotografias 2D, capturadas com uma única câmera, utilizadas na fotoantropometria, ainda são necessárias e esse tipo de material é mais acessível.

Cabe salientar ainda que as medidas faciais obtidas em imagens 2D podem ser subestimadas ou superestimadas em maior ou menor grau, de acordo com o ângulo e a norma de cada imagem, quando comparadas com as medidas faciais obtidas tridimensionalmente. Dessa forma, para fins de identificação humana, é recomendável que as medidas derivadas de imagens bidimensionais sejam analisadas individualmente e utilizadas com cautela, considerando-se as especificidades do ângulo e da norma da imagem utilizada, segundo a literatura^(36)^, sendo essa uma limitação a ser respeitada.

A análise facial numérica, baseada em medições e proporções, permite identificar as características morfológicas da face, incluindo o tamanho relativo das dimensões horizontais e verticais da face. Pesquisadores^(37)^ afirmaram que desvios dessas proporções podem indicar dimorfismos faciais ou condições clínicas específicas que requerem intervenção profissional. Pode-se inferir, portanto, que essa análise permite, ao especialista, refletir sobre as limitações terapêuticas que impactam negativamente nas funções estomatognáticas. Além disso, as assimetrias podem, segundo os autores supracitados^(37)^, afetar a percepção estética, a oclusão, o crescimento facial entre outros. Assim, quando a análise facial numérica foi procedida, foi realizada a seguir a verificação de convergências e divergências dos três métodos antropométricos faciais utilizados no presente estudo. Os resultados evidenciaram que, independentemente do método, as proporções não diferiram entre si, sem significância estatística, quando o terço foi o foco da análise, embora isso não tenha ocorrido em relação à proporção das hemifaces, devendo o profissional atentar-se a esse aspecto também, principalmente na prática pericial fonoaudiológica. Como não há pesquisas similares com os métodos utilizados, não foi possível discutir os presentes achados com a literatura.

No entanto, faz-se importante salientar que avaliações digitais envolvendo a face podem não ser confiáveis e, por esse motivo, recomenda-se que várias medições de distância e ângulos de imagens nas normas frontal e lateral, bem como a utilização de métodos combinados, como a fotoantropometria com a morfológica^(4)^ sejam utilizados para ampliar o julgamento profissional.

Assim, pode-se afirmar que na investigação tanto clínica quanto criminal é crucial que os métodos de análise sejam idênticos para garantir a consistência e a reprodutibilidade dos resultados. A confiança no método científico utilizado visa garantir que o sistema legal use informações forenses de forma precisa e válida, revelando que tal prática também precisa estar consubstanciada em evidências científicas.

No presente estudo foi verificado que as medidas e proporções foram maiores nos participantes do sexo masculino quando comparados aos do feminino, revelando dimorfismo sexual facial, como era esperado e já ratificado pela literatura. Na presente amostra, as diferenças reveladas pelo uso do paquímetro e pela aplicação do teste t de Student foram nas proporções Al-Al, Ex-Ch (tanto direito quanto esquerdo). Explica-se o resultado encontrado pelos resultados obtidos em pesquisa com crâneos secos a partir dos pontos forame infraorbital (direito e esquerdo) e o próstio, que evidenciaram o referido dimorfismo em amostra brasileira^(38)^.

As limitações do presente estudo foram o uso reduzido de pontos antropométricos e a utilização de um método que não faz a correção dos pixels. Essa limitação foi proposital a fim de verificar se a proposta seria robusta ou não para as práticas fonoaudiológicas que fazem uso da antropometria facial. As discordâncias entre os métodos induzem ao raciocínio de que as medidas diretas devem ser a prioridade de escolha, quando possíveis de serem obtidas e que, na prática pericial, perito e assistente técnico precisam utilizar o mesmo método (incluindo o *software* de análise) para a comparação entre as faces padrão e questionada, podendo-se hipotetizar que as fotografias podem gerar distorções que não retratam, necessariamente, a realidade.

Sugerem-se mais pesquisas relacionadas à antropometria facial com o *software* SAPO® a fim de ratificar ou não sua precisão para fins fonoaudiológicos, bem como comparar as medidas obtidas com diferentes marcas de câmeras fotográficas com circuitos fechados de televisão (CFTV) para validar ou não o uso da fotoantropometria na prática forense.

## CONCLUSÃO

Frente aos resultados obtidos, com a amostra estudada, pode-se concluir que:

- As medições diretas realizadas com o paquímetro foram diferentes das obtidas pelas fotoantropometria, com valores maiores pelo método direto.
- As medidas fotoantropométricas podem variar a depender do *software* utilizado, mesmo que utilizadas as mesmas técnicas de obtenção, calibração e medição (maior fragilidade de análise: terço médio da face, independentemente do fator sexo).

De forma adicional, foi constatado que as medidas antropométricas diretas ratificaram o dimorfismo sexual facial, uma vez que pessoas do sexo masculino apresentaram valores maiores do que as do sexo feminino, com exceção do terço médio.
